# Early development of pembrolizumab‐induced fulminant myositis and cardiotoxicity in a patient with metastatic thymoma

**DOI:** 10.1002/rcr2.1025

**Published:** 2022-08-24

**Authors:** Soo Yeon Jang, Sung Yong Lee, Hye Lim Lee, Juwhan Choi

**Affiliations:** ^1^ Division of Pulmonary, Allergy, and Critical Care Medicine, Department of Internal Medicine Korea University Guro Hospital, Korea University College of Medicine Seoul Republic of Korea; ^2^ Department of Neurology Korea University Guro Hospital, Korea University College of Medicine Seoul Republic of Korea

**Keywords:** immune checkpoint inhibitor, myositis, pembrolizumab, plasma exchange, thymoma

## Abstract

Herein, we report the case of a 48‐year‐old woman with metastatic thymoma who developed fulminant myositis with cardiotoxicity after one cycle of pembrolizumab treatment. She presented with severe muscle weakness and dyspnea, and her laboratory test results revealed increased muscle and cardiac enzyme levels. Despite an urgent initiation of systemic steroids, her muscle weakness and hypercapnia worsened, for which intravenous immunoglobulin G was initiated. However, hypercapnia did not improve, but the patient recovered completely after plasma exchange. Patients with thymic neoplasms could be susceptible to fulminant forms of immune‐related adverse effects because they lack normal thymic physiology. Clinicians must not hesitate to consider immunoglobulin G administration and plasma exchange therapy as the next treatment steps for steroid‐refractory patients.

## Graphical abstract

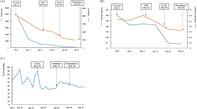

## INTRODUCTION

Immune checkpoint inhibitors (ICIs) increase the function of cytotoxic T lymphocytes in tumour cells. However, ICIs disrupt immune homeostasis and may cause an uncontrolled immune response, leading to immune‐related adverse effects (irAEs).^1^ IrAEs can occur throughout the body and be fatal when vital organs are involved. Pembrolizumab is an ICI that targets programmed cell death‐1 (PD‐1) receptors, and its common irAEs include dermatitis, colitis, pneumonitis, and thyroiditis.^2^ Severe irAEs such as myositis and cardiotoxicity are rare but significant because they can be fatal.^3^, ^4^ Here we report a case of pembrolizumab‐induced fulminant myositis with cardiotoxicity after one treatment cycle in a patient with metastatic thymoma.

## CASE REPORT

**FIGURE 1:**
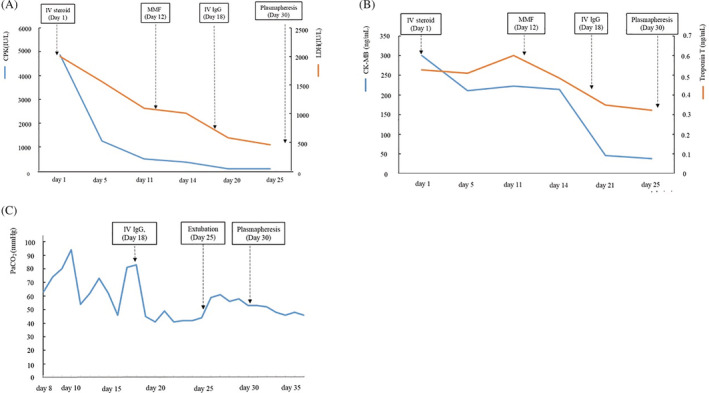
Blood chemistry and ABGA according to clinical course. (A) Muscle enzyme and LDH. (B) Cardiac enzyme. (C) PaCO_2_

A 48‐year‐old Korean woman with recurrent thymoma visited a clinic with motor weakness after the first cycle of pembrolizumab as 3rd line treatment. As the 1st line, etoposide‐carboplatin was given, and 2nd line cyclophosphamide, adriamycin, cisplatin and prednisolone was administered. She first experienced dyspnea and progressive ascending weakness in both legs 13 days after treatment with pembrolizumab. She could not even stand upright or raise her arms. On a neurologic examination, she showed medical research council grade 3 proximal weakness in the upper and lower limbs. Laboratory findings showed a dramatic increase in muscle and cardiac enzyme levels. Figure 1A,B shows the changes in levels of muscle and cardiac enzymes, respectively, as the treatment progressed. Electrocardiogram (ECG) showed t wave inversion in inferior leads, but echocardiogram was normal with normal range of N‐Terminal pro Brain Natriuretic Peptide (NT‐proBNP) level, indicative of preserved cardiac function. A nerve conduction study and electromyography showed myopathy in the proximal muscles of the upper and lower limbs but no peripheral neuropathy. She had a history of myasthenia gravis (MG) and thymectomy 13 years ago. After the surgery, she had few MG‐related symptom without any medication. Acetylcholine‐receptor‐binding antibody (Ach‐r‐binding Ab) titre was 6.17 nmol/L, which decreased from the last year, 7.62 nmol/L. Repetitive nerve stimulation tests (RNST) performed to screen for neuromuscular junction disorders showed negative findings.

She was administered intravenous methylprednisolone 2 mg/kg/day immediately after admission. Although the muscle enzyme levels consistently improved, her muscle weakness worsened despite 11 days of treatment with high‐dose methylprednisolone. Mycophenolate mofetil was added as an immunosuppressive agent on the 12th day after admission, but there was no effect. On the 18th day, immunoglobulin G (IVIgG) was administered intravenously. Respiratory failure progressed, and mechanical ventilation was applied. After 5 days of IVIgG therapy, the muscle enzyme levels recovered to the normal range. Her motor strength gradually improved from the lower extremities, and she was extubated on the 25th day. However, the weakness and hypercapnia worsened again after the extubation. Therefore, plasma exchange therapy was performed on the 30th day to remove the troublesome antibodies. After the therapy, her motor strength recovered, and she no longer complained of dyspnea.

## DISCUSSION

Thymoma is classified as thymic epithelial tumours (TETs) arising from the thymic epithelium. Studies have reported that TETs have high expression levels of PD‐L1, highlighting the therapeutic potential of ICIs for these tumours.^5^ In a normal thymus, T cell maturation occurs, and T cell precursors that react against tissue‐specific self‐antigens undergo apoptosis. This important process, called negative selection, results in immune tolerance. TETs cause problems in this process, resulting in autoreactive T cells entering the circulation. Therefore, patients with TET are more susceptible to irAEs than are patients with other cancers.^5^

Myositis, a known autoimmune side effect of ICI usage, occurs in 1% of patients treated with anti‐PD‐1 antibodies.^3^ Remarkably, 8% of patients with thymic carcinoma treated with pembrolizumab show myositis. Cardiotoxicity, the most common type being myocarditis, is also rare but can be fatal. Among patients with cancers other than TET treated with ICIs, less than 1% develop myocarditis, whereas 5% of patients with thymic carcinoma treated with ICIs present with myocarditis.^5^ Our patient developed fulminant myositis with myocarditis within 2 weeks after initiation of treatment with pembrolizumab. Its early development was notable because irAEs usually occur within a few weeks to months.^6^ She had prior 1st and 2nd line chemotherapy, which are not common agents causative of cardiotoxicity. Before treatment with pembrolizumab, there was no cardiac symptom with normal ECG, which indicate that prior treatment had little effect on cardiac toxicity.

We consulted the patient to a neurologist and constantly discussed the case to exclude the possibility of MG relapse. Although she had a history of MG, she has had no symptom without any medication after the surgery. RNST showed negative findings, and Ach‐r‐binding Ab titre was diminished. We tried pyridostigmine suspecting MG aggravation when she was admitted, but there was no effect. Steroid also had no effect on motor weakness. Instead, muscle and cardiac enzyme level decreased with steroid treatment. Therefore, we concluded the case as myositis than MG relapse.

Close monitoring for the early diagnosis of irAEs is crucial to prevent progression to fatal complications. No symptoms must be neglected, although they are sometimes confused with cancer‐related symptoms.^7^ The rapid initiation of treatment is important with active consultation to multidisciplinary specialists, and practitioners must monitor the treatment response. When systemic steroids seem ineffective, IVIgG administration and plasma exchange therapy must be considered as the next treatment steps.

## AUTHOR CONTRIBUTION

Soo yeon Jang wrote and edited the manuscript. Sung Yong Lee critically revised the manuscript. Hye Lim Lee revised the manuscript. Juwhan Choi reviewed and approved the final manuscript.

## CONFLICT OF INTEREST

None declared.

## ETHICS STATEMENT

The authors declare that appropriate written informed consent was obtained for the publication of this manuscript and accompanying image.

## Data Availability

The data that support the findings of this study are available on request from the corresponding author.
